# Increased neutrophil count Is associated with the development of chronic kidney disease in patients with diabetes

**DOI:** 10.1111/1753-0407.13292

**Published:** 2022-07-04

**Authors:** Rui Zhang, Jin Chen, Yanqin Xiong, Lihong Wang, Xinmei Huang, Tiange Sun, Bingbing Zha, Yueyue Wu, Cuili Yan, Shufei Zang, Qin Zhou, Zhe Huang, Jun Liu

**Affiliations:** ^1^ Department of Endocrinology Shanghai Fifth People's Hospital, Fudan University Shanghai China; ^2^ MaQiao Community Health Service Center Shanghai China; ^3^ Gumei Community Health Service Center Shanghai China; ^4^ Kidney Disease Center, the First Affiliated Hospital, College of Medicine Zhejiang University Hangzhou China; ^5^ Key Laboratory of Disease Prevention and Control Technology Hangzhou China; ^6^ National Key Clinical Department of Kidney Disease Hangzhou China; ^7^ Institute of Nephrology Zhejiang University Hangzhou China; ^8^ The Third Grade Laboratory under the National State, Administration of Traditional Chinese Medicine Hangzhou China; ^9^ Department of Genetics and Developmental Science School of Life Sciences and Biotechnology, Shanghai Jiao Tong University Shanghai China

**Keywords:** chronic kidney disease, diabetes, inflammation, neutrophils, 慢性肾脏疾病, 糖尿病, 炎症, 中性粒细胞

## Abstract

**Background:**

This study aims to investigate the potential association of peripheral inflammatory blood cell parameters with the incidence and progression of chronic kidney disease (CKD) in patients with diabetes.

**Methods:**

The cross‐sectional study included 1192 subjects with diabetes derived from one center. The cohort study included 2060 subjects with diabetes derived from another two centers followed up for 4 years. Logistic regression and Cox proportional hazards models were used to evaluate the association of peripheral inflammatory blood cell with CKD.

**Results:**

In the cross‐sectional study, neutrophil count performed best as an independent risk factor for CKD (odds ratio 2.556 [95% confidence interval 1.111, 5.879]) even after 1:1 case–control matching for age, gender, history of high blood pressure and duration of diabetes. Spline regression revealed a significant linear association of CKD incidence with continuous neutrophil count in excess of 3.6 × 10^9^/L. In the cohort study, subjects were grouped based on tertile of neutrophil count and neutrophil‐to‐lymphocyte ratio. Cox regression analysis results showed that only neutrophil count was independently associated with CKD progression (the highest group vs. the lowest group, hazard ratio 2.293 [95% confidence interval 1.260, 4.171]) after fully adjusting for potential confounders. The cumulative incidence of CKD progression in patients with diabetes gradually increased with increasing neutrophil count (53 (7.7%) subjects in the lowest group vs. 60 (8.2%) in the middle group vs. 78 (12.2%) in the highest group).

**Conclusions:**

This study suggested that neutrophil count is an independent risk factor for progression of CKD in patients with diabetes.

## Graphical abstract

Highlights

Neutrophil count was an independent risk factor for CKD in patients with diabetes, especially when it exceeded 3.6 × 10^9^/L, and increased neutrophil count could predict progression of CKD in patients with diabetes.

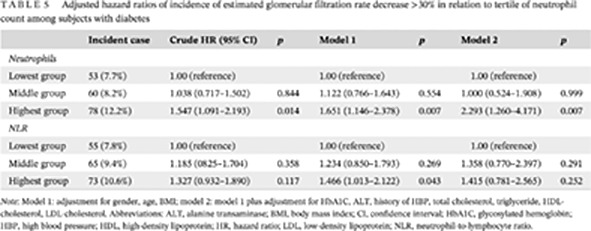

## 摘要

**背景:**

本研究旨在探讨糖尿病人中外周血炎性细胞参数与慢性肾脏疾病(CKD)的发生和发展之间的潜在相关性。

**方法:**

以1192名来自同一研究中心的受试者作为横断面研究。来自另外两个中心的2060名受试者随访4年，进入队列研究。采用Logistic回归和COX比例风险模型评价外周血炎性细胞与CKD的相关性。

**结果:**

在横断面研究中，即使在年龄、性别、高血压病史和糖尿病病程进行1:1病例对照匹配后，中性粒细胞计数仍然是CKD的最相关的独立危险因素(OR 2.556[95%CI 1.111，5.879])。样条回归显示CKD发病率与中性粒细胞计数持续超过3.6×10^9^/L呈显著线性相关。在队列研究中，受试者根据中性粒细胞计数和中性粒细胞/淋巴细胞比率的三分位进行分组。COX回归分析结果显示，在完全校正潜在混杂因素后，只有中性粒细胞计数与CKD进展独立相关(最高组vs.最低组，HR 2.293[95%CI 1.260，4.171])。随着中性粒细胞计数的增加，糖尿病患者CKD进展的累积发生率逐渐增加，最低组53例(7.7%)，中间组60例(8.2%)，最高组78例(12.2%)。

**结论:**

本研究提示中性粒细胞计数是糖尿病患者CKD进展的独立危险因素。

## 1 INTRODUCTION

The global prevalence of diabetes mellitus has increased enormously over the past few decades. Approximately half of all affected patients will develop chronic kidney disease (CKD).^1^ Of the microvascular and macrovascular complications of diabetes, CKD imposes a high financial burden. Cardiovascular mortality and progression to end‐stage renal disease are the two major adverse health outcomes in patients with diabetes and CKD.^2^ Consequently, prevention or management of CKD is the primary aim of management in patients with diabetes. Nonetheless current management that consists of controlling risk factors such as hyperglycemia, hypertension, hyperuricemia, and lipid regulation do not prevent the progression of nephropathy. Therefore, exploring the pathophysiology of CKD in diabetes is crucial to its management.

An increasing number of studies has indicated that the pathogenesis of CKD in diabetes is multifactorial, and low‐grade chronic inflammation plays a crucial role.^3^, ^4^, ^5^, ^6^ Routine inflammatory blood cell parameters such as white blood cell (WBC) count, neutrophil count, and neutrophil‐to‐lymphocyte ratio (NLR) have been investigated for an association with and ability to predict diabetes and CKD.^7^, ^8^, ^9^, ^10^ Nonetheless results have been inconsistent, possibly due to small sample sizes and a lack of well‐characterized study cohorts.

This study aimed to investigate the potential association of peripheral inflammatory blood cell parameters with the incidence and progression of CKD in patients with diabetes by cross‐sectional study of expanded sample size and cohort study. First, in the cross‐sectional study, WBC count, neutrophil count and NLR were all increased and lymphocyte count was decreased in patients with CKD. All were also associated with parameters of nephropathy including creatinine (Cr), estimated glomerular filtration rate (eGFR) and urine albumin/creatinine ratio (UACR). Nonetheless neutrophil count was the best independent risk factor for and predictor of CKD. In addition, significant linear associations between continuous neutrophil count, NLR and the incidence of CKD were analyzed by spline regression. Finally, our cohort study further confirmed that an increased neutrophil count, not NLR, predicted onset of CKD in diabetes. Moreover, there was a linear relationship between increasing neutrophil count and cumulative incidence of CKD.

## 2 METHODS

### 2.1 Study population

**FIGURE 1:**
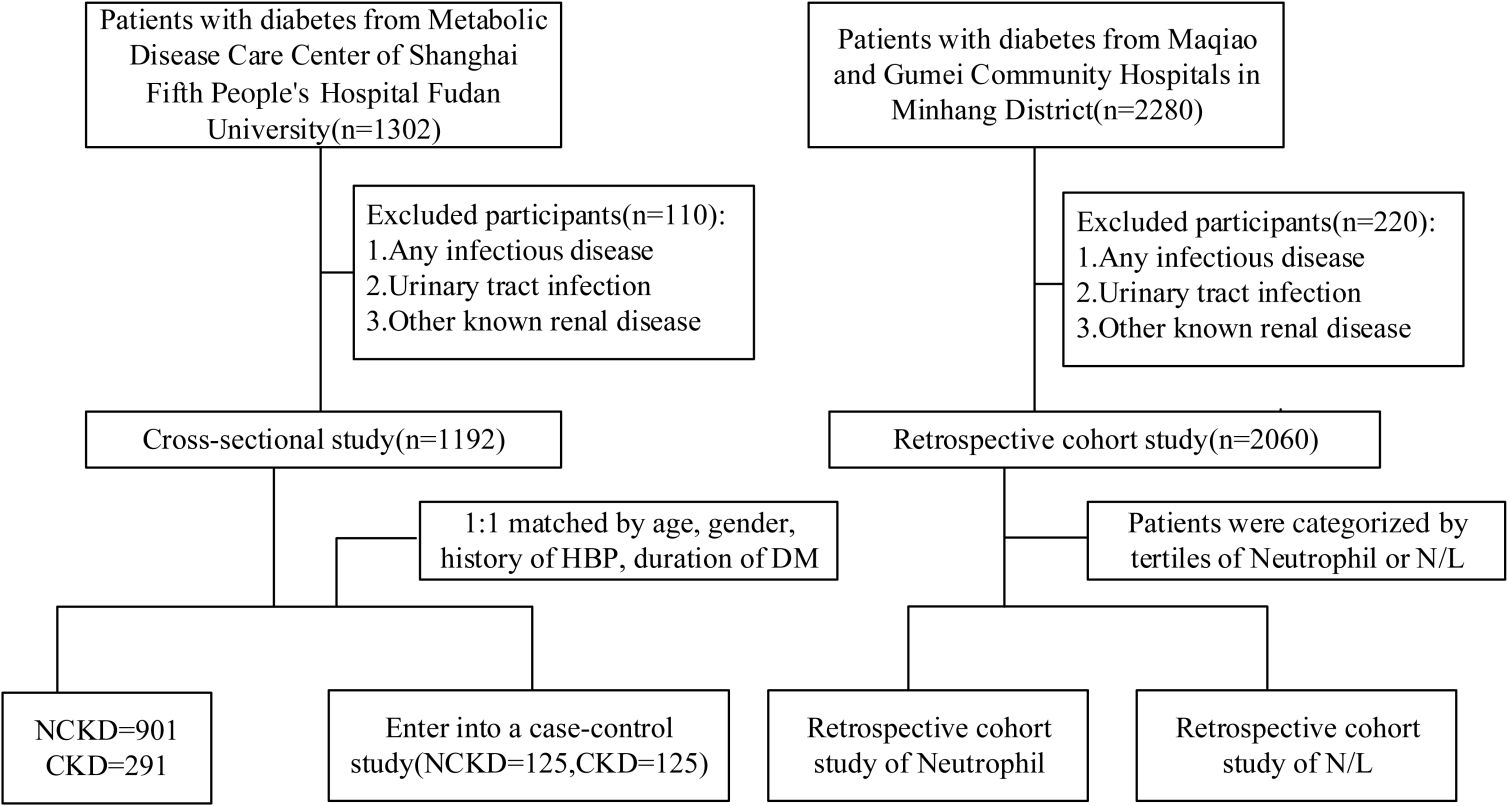
Flow chart of this study. CKD, chronic kidney disease; DM, diabetes mellitus; HBP, high blood pressure; NCKD, without chronic kidney disease; N/L, netrophil‐to‐lymphocyte ratio

A total of 3582 patients with diabetes were screened, of whom 3252 entered the study. A total of 1192 patients with diabetes (291 with CKD and 901 without CKD [NCKD]) who attended the Metabolic Disease Care Center of Shanghai Fifth People's Hospital Fudan University from May 2017 to July 2019 served as the cross‐sectional study. Another 2060 patients with diabetes were recruited into the cohort study, who were followed up annually for 4 years from January 2016 to December 2019 at Maqiao and Gumei Community Hospitals in Minhang District, Shanghai. This analysis involving human participants was approved by the Independent Ethics Committee of the Fifth People's Hospital of Shanghai, Fudan University (2010–2024). Consent has been obtained from each patient or subject after full explanation of the purpose and nature of all procedures used. The study procedure is described in Figure 1.

#### 2.1.1 Definition of diabetes

Diagnosis of diabetes was based on the 2019 Chinese Diabetes Society criteria: (1) typical symptoms of diabetes (polydipsia, polyuria, polyphagia, and weight loss) plus random plasma glucose ≥11.1 mmol/L, (2) fasting plasma glucose ≥7.0 mmol/L, or (3) oral glucose tolerance test 2hPG ≥ 11.1 mmol/L.

#### 2.1.2 EGFR calculation, definition of CKD group, and CKD progression

Equations to estimate GFR use serum creatinine and a combination of age, gender, ethnicity, and body size as surrogates for the non‐GFR determinants of serum creatinine. We used the modified Modification of Diet in Renal Disease equations for use in individuals from China and Japan that have been reported previously. In cross‐sectional study, definition of CKD is based on the presence of decreased kidney function (ie, GFR <60 ml/min per 1·73 m^2^) or UACR ≥ 30 mg/g (two of three specimens of UACR collected within a 3‐ to 6‐ month period).^11^ In the cohort study, CKD progression was defined in the cohort as 30% decline in eGFR.

Participants were excluded for any of the following reasons: (1) presence of any infectious disease excluding by medical history collection and routine examination such as chest X‐ray, electrocardiogram, and biochemistry test; (2) presence of viral infection or positive carrier status (hepatitis B virus, syphilis or HIV); (3) previous diagnosis of urinary tract infection, urolithiasis, or liver cirrhosis; and (4) other known renal disease: IgA nephropathy, nephrotic syndrome. All study subjects were of Han Chinese origin and lived in the same region at the time of the study.

### 2.2 Data collection and laboratory assessments

All subjects underwent blood and urine sample analyses. After an overnight fast for 12 h, blood samples were collected for blood cell counts (Automatic Blood cell analyzer, Sysmex XN9000, Japan) and measurement of biochemical parameters (Automatic biochemical analyzer, Roche Cobas 8000, Switzerland). A urine sample was collected for assessment of UACR. Anthropometric parameters were used to calculate body mass index (BMI), and blood pressure was measured with a digital automatic blood pressure monitor (model HEM‐907; Omron, Tokyo, Japan). A patient questionnaire was completed and included information about history of high blood pressure (HBP) and duration of diabetes.

### 2.3 Statistical analysis

Data are shown as mean ± SD, unless otherwise noted. *T*‐test was used for between‐group comparisons of continuous variables and the *χ* ^*2*^ test for categorical variables. Skewed variables are expressed as median (interquartile range), and rank sum test was used to compare the difference between two groups. To avoid potential bias due to uneven distribution of covariates between type 2 patients with or without CKD, a case–control matching method was employed to match variables that included gender, age, history of HBP and duration of diabetes. Matching tolerance was 0, 2, 0, and 0 respectively. Logistic regression analysis was performed to detect the predictability of WBC, neutrophil and lymphocyte count, and NLR for CKD. The covariates were prespecified for inclusion in multivariable models based on clinical and biologic importance. A continuous association of neutrophil count and NLR with CKD incidence was determined by spline regression analysis.

In the retrospective cohort study, patients were divided into three groups based on tertile of neutrophil count: lowest group (<3.19 × 10^9^/L), middle group (3.19–4.10 × 10^9^/L), and highest group (>4.10 × 10^9^/L); and NLR: lowest group (<1.58), middle group (1.58–2.16), and highest group (>2.16). Analysis of variance (ANOVA) and Student's *t‐test* were used to identify difference in mean between groups. Variables with a skewed distribution were analyzed by Wilcoxon tests. The cumulative incidence of CKD in relation to neutrophil tertile was assessed by Kaplan–Meier plots.

All statistical analyses were performed using SPSS 25.0 software (IBM SPSS Inc, Chicago, IL, USA). All *p* values are two tailed, and statistical significance was set at *p* < 0.05.

## 3 RESULTS

### 3.1 Characteristics of patients with CKD and without CKD in all subjects and matched subjects

Clinical characteristics of the patients were stratified according to their nephropathy status (Table 1). A total of 1192 patients with diabetes were included in this cross‐sectional study, among whom 24.4% had kidney disease, as defined by eGFR <60%, or UACR ≥ 300 mg/g. Female patients with older age and diabetes duration longer than 10 years were more likely to develop kidney disease (*p* < 0.001). Compared with those without CKD, patients with CKD were more likely to have hypertension, higher systolic blood pressure (SBP), diastolic blood pressure (DBP), uric acid (UA), and UACR (all *p* < 0.001). There was no significant difference in BMI, fasting glucose, glycosylated hemoglobin (HbA1c), total cholesterol (TC), high‐density lipoprotein cholesterol (HDL‐c), and low‐density lipoprotein cholesterol (LDL‐c) between the two groups (*p* > 0.05). The WBC count (6.74 ± 0.13 vs. 6.41 ± 0.06 × 10^9^/L, *p* < 0.001), neutrophil count (4.36 ± 0.11 vs. 3.68 ± 0.05 × 10^9^/L, *p* < 0.001), and NLR (2.86 ± 0.11 vs. 2.08 ± 0.04, *p* < 0.001) were much higher in patients with CKD than those without, whereas the lymphocyte count was much lower in patients with CKD (1.76 ± 0.04 vs. 1.94 ± 0.02 × 10^9^/L, *p* < 0.001).

**TABLE 1:** Clinical characteristics of all NCKD and CKD subjects and matched case–control study

	All subjects			Matched case–control study		
	NCKD	CKD	*p*	NCKD	CKD	*p*
*n*	901	291	‐	125	125	‐
Sex (male) (*n*/%)	522 (57.9%)	136 (46.9%)	0.001	68 (48.6%)	68 (48.6%)	1.000
Age (years)	58.54 ± 0.44	70.55 ± 0.64	<0.001	67.87 ± 0.84	68.01 ± 0.86	0.289
Duration of diabetes (>10 years/%)	207 (48.5%)	128 (78.5%)	<0.001	79 (63.2%)	79 (63.2%)	1.000
BMI (kg/m^2^)	25.09 ± 0.10	25.29 ± 0.20	0.385	24.43 ± 0.32	25.07 ± 0.31	0.152
SBP (mmHg)	130 (120–140)	136 (124–150)	<0.001	133 (120–140)	135 (120–143.5)	0.289
DBP (mmHg)	80 (72–86)	78 (70–83)	0.008	78 (70–83)	79 (70–82)	0.999
HBP (%)	299 (33.2%)	151 (51.9%)	<0.001	79 (63.2%)	79 (63.2%)	1.000
TC (mmol/L)	4.38 ± 0.04	4.39 ± 0.08	0.893	4.12 ± 0.11	4.38 ± 0.13	0.089
TG (mmol/L)	1.5 (1.05–2.28)	1.66 (1.19–2.47)	0.025	1.36 (0.95–2.10)	1.60 (1.19–2.39)	0.016
HDL (mmol/L)	1.01 (0.84–1.22)	0.98 (0.82–1.24)	0.321	1.04 (0.83–1.27)	0.97 (0.80–1.20)	0.203
LDL (mmol/L)	2.79 ± 0.03	2.77 ± 0.07	0.715	2.62 ± 0.10	2.69 ± 0.11	0.616
Cr (mmol/L)	62 (52–74)	103 (81–130)	<0.001	62 (52–75)	102 (82–133)	<0.001
UA (mmol/L)	305.83 ± 3.14	375.62 ± 6.58	<0.001	291.03 ± 8.42	368.04 ± 10.14	<0.001
eGFR (%)	106.70 ± 0.96	44.31 ± 0.78	<0.001	89.56 (76.7–111.91)	51.4 (39.49–58.82)	<0.001
UACR (mg/g)	9 (5–26)	111 (27–711)	<0.001	19 (5–54)	96 (25–739)	<0.001
HbA1C (%)	8.93 ± 0.07	9.06 ± 0.14	0.377	8.67 ± 0.18	8.70 ± 0.20	0.911
FBG (mmol/L)	7.88 ± 0.10	7.45 ± 0.19	0.029	7.52 ± 0.35	7.80 ± 0.34	0.553
2hBG (mmol/L)	12.80 ± 0.17	12.50 ± 0.31	0.401	13.79 ± 0.57	13.12 ± 0.59	0.375
WBC (×10^9^/L)	6.41 ± 0.06	6.74 ± 0.13	0.018	6.20 ± 0.16	6.59 ± 0.20	0.139
Neutrophils (×10^9^/L)	3.68 ± 0.05	4.36 ± 0.11	<0.001	3.71 ± 0.13	4.27 ± 0.17	0.011
Lymphocytes (×10^9^/L)	1.94 ± 0.02	1.76 ± 0.04	<0.001	1.78 ± 0.06	1.73 ± 0.05	0.481
NLR	2.08 ± 0.04	2.86 ± 0.11	<0.001	2.36 ± 0.12	2.79 ± 0.15	0.035

*Note*: Data are mean ± SE, median (interquartile range) or *n* (%).

Abbreviations: BMI, body mass index; CKD, chronic kidney disease; Cr, creatinine; DBP, diastolic blood pressure; eGFR, estimated glomerular filtration rate; FBG, fasting blood glucose; HbA1C, glycosylated hemoglobin; HBP, high blood pressure; HDL, high‐density lipoprotein; LDL, low‐density lipoprotein; NCKD, without chronic kidney disease; NLR, neutrophil‐to‐lymphocyte ratio; SBP, systolic blood pressure; TC, total cholesterol; TG, triglyceride; UA, uric acid; UACR, urinary albumin‐to‐creatinine ratio; WBC, white blood cell; 2hBG, blood glucose 2 h after oral glucose load.

A 1:1 case–control matching procedure (matching for gender, age, history of HBP, and duration of diabetes) was performed to avoid the potential bias of covariates that were not evenly distributed between patients with and without CKD. After matching, patients with higher triglycerides (TG) were still more likely to develop nephropathy. Furthermore, there remained higher WBC count (6.59 ± 0.20 vs. 6.20 ± 0.16 × 10^9^/L, *p* = 0.139), neutrophil count (4.27 ± 0.17 vs. 3.71 ± 0.13 × 10^9^/L, *p* = 0.011), and NLR (2.79 ± 0.15 vs. 2.36 ± 0.12, *p* = 0.035) but lower lymphocyte count (1.73 ± 0.05 vs. 1.78 ± 0.06 × 10^9^/L, *p* = 0.481) in patients with CKD compared with those without (Table 1).

### 3.2 Increased WBC and neutrophil count and NLR were associated with Cr, eGFR, and UACR

**FIGURE 2:**
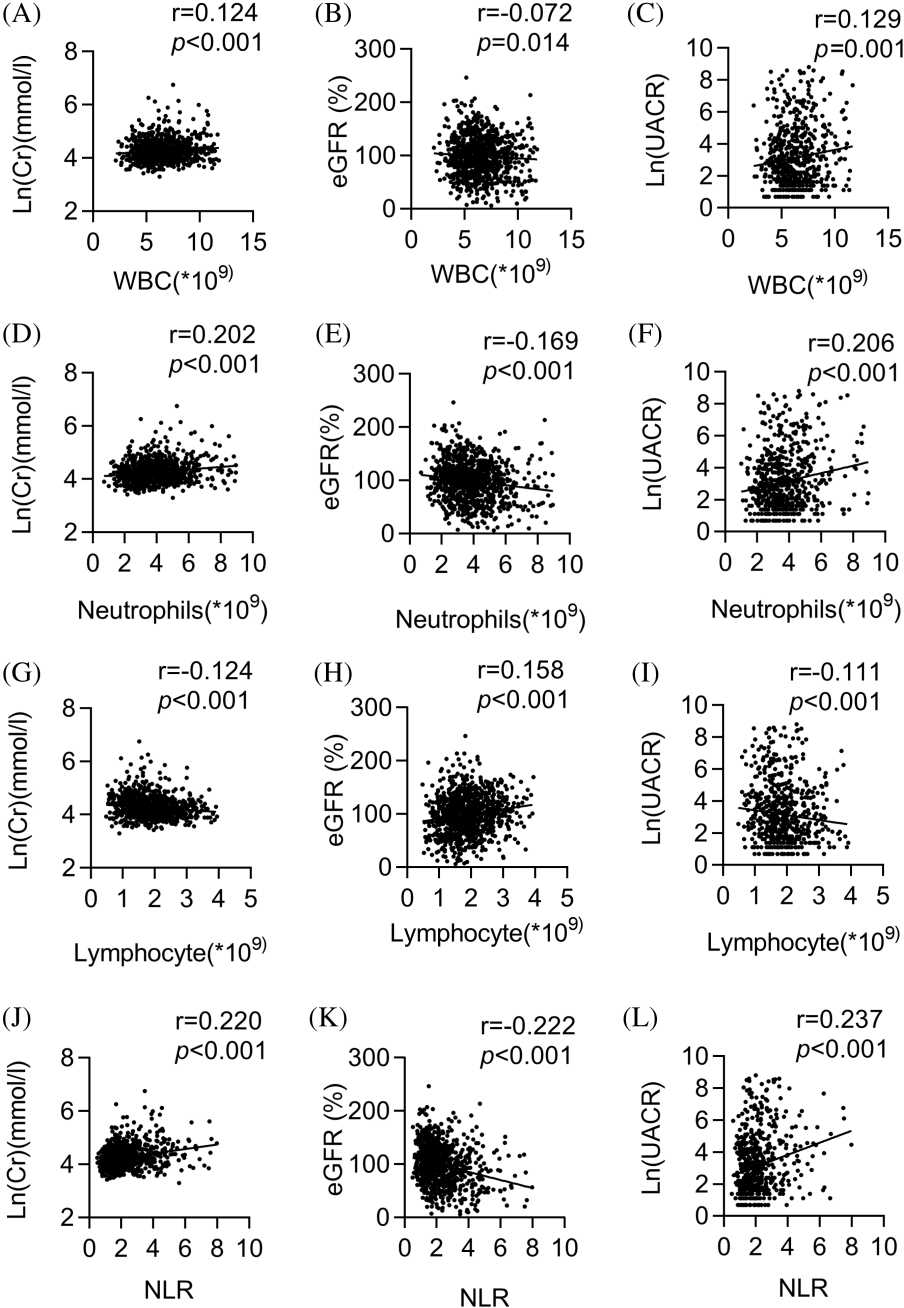
Association of WBC, neutrophil, and lymphocyte count and NLR with Cr, eGFR, and UACR. Association of WBC, neutrophil, and lymphocyte count and NLR with Cr, eGFR, and UACR. WBC count was positively associated with Cr (*r* = 0.124, *p* < 0.001) (A) and UACR (*r* = 0.129, *p* = 0.001) (C) and negatively associated with eGFR (*r* = −0.072, *p* = 0.014) (B). There was also a strong association between neutrophil count, Cr (*r* = 0.202, *p* < 0.001) (D) and UACR (*r* = 0.206, *p* < 0.001) (F) and a negative association with eGFR (*r* = −0.169, *p* < 0.001) (E). Additionally, NLR exhibited a significant and positive association with Cr (*r* = 0.220, *p* < 0.001) (J) and UACR (*r* = 0.237, *p* < 0.001) (L), but a negative association with eGFR (*r* = −0.222, *p* < 0.001) (K). Cr, creatinine; eGFR, estimated glomerular filtration rate; NLR, neutrophil‐to‐lymphocyte ratio; UA, uric acid; UACR, urinary albumin‐to‐creatinine ratio; WBC, white blood cell. e

Simple linear regression analyses were performed to investigate the correlation between CKD and biochemical and inflammatory blood cell parameters in patients with diabetes. Level of creatinine was positively associated with age, BMI, TG, and UA (all *p* < 0.05) and negatively associated with HDL (*p* < 0.05). There was also a strong association between UACR and age, SBP, UA, TC, TG, and LDL (all *p* < 0.05), whereas eGFR was negatively associated with age, SBP, DBP, BMI, and UA (all *p* < 0.001) (Table 2). There was a significant and positive association of WBC count, neutrophil count, and NLR with Cr and UACR (all *p* < 0.001), but a negative association with eGFR (all *p* < 0.001). Additionally, lymphocyte count was negatively associated with Cr and positively associated with eGFR (all *p* < 0.05) (Table 2, Figure 2).

**TABLE 2:** Association of Cr, eGFR, and UACR with biochemical and inflammatory blood cell parameters

	Ln (Cr)		eGFR		Ln (UACR)	
	*r*	*p*	*r*	*p*	*r*	*p*
Age (years)	0.237	<0.001	−0.449	<0.001	0.147	<0.001
Ln (SBP) (mmHg)	0.066	0.022	−0.097	0.001	0.251	<0.001
Ln (DBP) (mmHg)	−0.019	0.515	0.065	0.025	0.054	0.164
BMI (kg/m^2^)	0.154	<0.001	−0.16	<0.001	0.029	0.489
HbA1C (mmol/L)	−0.035	0.263	0.115	<0.001	0.091	0.026
UA (mmol/L)	0.459	<0.001	−0.38	<0.001	0.234	<0.001
TC (mmol/L)	−0.042	0.173	0.069	0.024	0.163	<0.001
Ln (TG) (mmol/L)	0.083	0.007	−0.038	0.213	0.16	<0.001
Ln (HDL) (mmol/L)	−0.118	<0.001	0.039	0.204	−0.008	0.842
LDL (mmol/L)	−0.043	0.157	0.061	0.047	0.118	0.002
WBC (×10^9^/L)	0.124	<0.001	−0.072	0.014	0.129	0.001
Neutrophils (×10^9^/L)	0.202	<0.001	−0.169	<0.001	0.206	<0.001
Lymphocytes (×10^9^/L)	−0.124	<0.001	0.158	<0.001	−0.111	0.004
NLR	0.220	<0.001	−0.222	<0.001	0.237	<0.001

Abbreviations: BMI, body mass index; Cr, creatinine; DBP, diastolic blood pressure; eGFR, estimated glomerular filtration rate; HbA1C, glycosylated hemoglobin; HDL, high‐density lipoprotein; LDL, low‐density lipoprotein; NLR, neutrophil‐to‐lymphocyte ratio; SBP, systolic blood pressure; TC, total cholesterol; TG, triglyceride; UA, uric acid; UACR, urinary albumin‐to‐creatinine ratio; WBC, white blood cell.

### 3.3 Increased neutrophil count was associated with CKD both in all subjects and 1:1 matched subjects, whereas NLR was associated with CKD in all subjects

To estimate the association of WBC count, neutrophil count, and NLR with CKD development in patients with diabetes, logistic regression analysis with enter selection was performed separately in all subjects and in the matched subjects. In all subjects, after adjusting for age, gender, BMI, duration of diabetes, history of HBP, HbA1c, UA, TC, TG, HDL and LDL, WBC (odds ratio [OR] = 2.163; 95% confidence interval [CI], 1.080–4.332 in the highest tertile vs. the lowest tertile, *p* = 0.030), neutrophil count (OR = 2.102; 95% CI, 1.059–4.172 in the highest tertile vs. the lowest tertile, *p* = 0.034) and NLR (OR = 2.347; 95% CI, 1.198–4.597 in the highest tertile vs. the lowest tertile, *p* = 0.013) were independently associated with CKD development but not lymphocyte count (Table 3). In the matched subjects, after adjusting for BMI, duration of diabetes, HbA1c, UA, TC, TG, and HDL and LDL level, neutrophil count, not NLR, remained closely associated with development of CKD (OR = 2.556; 95% CI, 1.111–5.879 in the highest tertile vs. the lowest tertile, *p* = 0.027) (Table 3).

**TABLE 3:** Logistic regression analysis (enter method) to determine the risk factors for nephropathy in the study compared with NCKD

	OR (95% CI)	*p*	OR	*p*
WBC				
*Gender*				
Male	Reference			
Female	1.268 (0.732–2.195)	0.398		
*Age (years)*	1.103 (1.072–1.134)	<0.001		
*BMI (kg/m* ^*2*^ *)*				
<25	Reference			
≥25	0.443 (0.249–0.788)	0.006	0.652 (0.331–1.285)	0.217
*Duration of diabetes*				
<10 years	Reference			
>10 years	3.374 (1.825–6.238)	<0.001		
*HBP*				
No	Reference			
Yes	1.318 (0.760–2.287)	0.326		
HbA1C (%)	1.066 (0.928–1.225)	0.366	1.087 (0.925–1.277)	0.313
UA (mmol/L)	1.01 (1.006–1.013)	<0.001	1.008 (1.004–1.012)	<0.001
TC (mmol/L)	1.76 (0.711–4.354)	0.221	0.936 (0.525–1.669)	0.823
TG (mmol/L)	0.99 (0.686–1.431)	0.959	2.101 (0.407–10.839)	0.375
HDL (mmol/L)	0.555 (0.176–1.75)	0.315	0.642 (0.125–3.3)	0.595
LDL (mmol/L)	0.607 (0.250–1.471)	0.268	0.504 (0.098–2.603)	0.413
Tertile of WBC count (×10^9^/L)				
First	Reference		Reference	
Second	1.244 (0.652–2.373)	0.509	1.147 (0.527–2.498)	0.730
Third	2.163 (1.080–4.332)	0.030	2.461 (1.069–5.665)	0.034
N + L				
*Gender*				
Male	Reference			
Female	1.293 (0.731–2.287)	0.377		
Age (years)	1.103 (1.071–1.135)	<0.001		
*BMI (kg/m* ^*2*^ *)*				
<25	Reference			
≥25	0.506 (0.285–0.896)	0.019	0.717 (0.366–1.405)	0.333
*Duration of diabetes*				
<10 years	Reference			
≥10 years	3.113 (1.666–5.818)	<0.001		
*HBP*				
No	Reference			
Yes	1.213 (0.690–2.134)	0.502		
HbA1C (%)	1.111 (0.963–1.283)	0.15	1.104 (0.937–1.302)	0.238
UA (mmol/L)	1.01 (1.007–1.013)	<0.001	1.008 (1.004–1.012)	<0.001
TC (mmol/L)	1.637 (0.600–4.463)	0.336	0.984 (0.547–1.767)	0.956
TG (mmol/L)	0.999 (0.672–1.485)	0.995	1.783 (0.345–9.221)	0.490
HDL (mmol/L)	0.554 (0.166–1.842)	0.335	0.687 (0.134–3.516)	0.652
LDL (mmol/L)	0.695 (0.259–1.867)	0.471	0.609 (0.117–3.175)	0.556
*Tertile of neutrophil count (×10* ^*9*^ */L)*				
First	Reference		Reference	
Second	1.328 (0.680–2.593)	0.406	1.738 (0.777–3.885)	0.178
Third	2.102 (1.059–4.172)	0.034	2.556 (1.111–5.879)	0.027
*Tertile of lymphocyte count (×10* ^*9*^ */L)*				
First	Reference		Reference	
Second	1.067 (0.564–2.021)	0.841	1.126 (0.504–2.512)	0.773
Third	0.944 (0.455–1.957)	0.876	0.876 (0.382–2.010)	0.755
NLR				
*Gender*				
Male	Reference			
Female	1.277 (0.734–2.223)	0.387		
*Age (years)*	1.1 (1.069–1.132)	<0.001		
*BMI (kg/m* ^*2*^ *)*				
<25	Reference			
≥25	0.52 (0.299–0.902)	0.020	0.783 (0.409–1.498)	0.460
*Duration of diabetes*				
<10 years	Reference			
>10 years	3.076 (1.672–5.658)	<0.001		
*HBP*				
No	Reference			
Yes	1.297 (0.746–2.256)	0.356		
HbA1C (%)	1.113 (0.969–1.278)	0.130	1.122 (0.958–1.314)	0.154
UA (mmol/L)	1.009 (1.006–1.012)	<0.001	1.007 (1.004–1.011)	<0.001
TC (mmol/L)	1.351 (0.503–3.631)	0.551	1.132 (0.640–2.000)	0.671
TG (mmol/L)	1.12 (0.759–1.652)	0.569	1.376 (0.273–6.918)	0.699
HDL (mmol/L)	0.664 (0.203–2.175)	0.499	0.895 (0.175–4.584)	0.894
LDL (mmol/L)	0.818 (0.309–2.170)	0.687	0.787 (0.154–4.017)	0.774
*NLR*				
First	Reference		Reference	
Second	1.391 (0.704–2.745)	0.342	1.269 (0.596–2.701)	0.537
Third	2.347 (1.198–4.597)	0.013	1.899 (0.843–4.279)	0.122

Abbreviations: BMI, body mass index; HbA1C, glycosylated hemoglobin; HBP, high blood pressure; HDL, high‐density lipoprotein; LDL, low‐density lipoprotein; NCKD,without chronic kidney disease; NLR, neutrophil‐to‐lymphocyte ratio; OR, odds ratio; TC, total cholesterol; TG, triglyceride; UA, uric acid; WBC, white blood cell.

### 3.4 Spline regression: Continuous neutrophil count and NLR were both associated with the incidence of CKD in patients with diabetes

**FIGURE 3:**
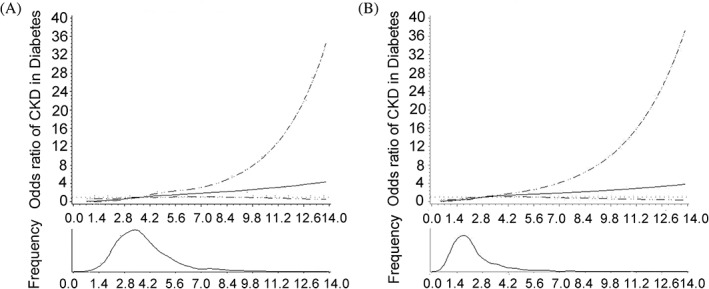
Continuous association of neutrophil count and NLR with the incidence of CKD in diabetes. Adjusted for BMI and gender. A for neutrophil count; B for NLR. The risk of developing CKD in diabetic patients increased when neutrophil count exceeded 3.6 × 10^9^/L and NLR exceeded 2.8. BMI, body mass index; CKD, chronic kidney disease; NLR, neutrophil‐to‐lymphocyte ratio

After adjusting for gender and BMI, two spline regression models showed a significant relationship between continuous neutrophil count and NLR, and the incidence of CKD. The risk of developing CKD in patients with diabetes increased when neutrophil count exceeded 3.6 × 10^9^/L and NLR exceeded 2.8 (Figure 3).

### 3.5 Increased neutrophil count predicted the development of CKD in the retrospective cohort study

As a retrospective cohort study 2060 patients with diabetes were recruited from two other centers. First, we analyzed dynamics of neutrophils and NLR, with reference parameters including mean, standard deviation, and coefficient of variation (CV). We found that the mean value of neutrophils and NLR was relatively stable during the 4 years, and the CV were all less than 25% and 30% respectively (Table S1 and Figure S1).

All subjects were stratified at baseline into one of three groups according to tertile of neutrophil count and NLR. Across the lowest, middle, and highest groups of neutrophil tertile, there was a stepwise increase in the proportion of male patients (42.5%, 46.0%, 50.0%, *p* = 0.024) and patients with HBP (76.7%, 82.2%, 82.0%, *p* = 0.037), as well as the level of TG, Cr, fasting glucose, and HbA1c (all *p* < 0.001) and a stepwise decrease in the eGFR and HDL level (both *p* < 0.05). Across the lowest, middle and highest groups of NLR, there was also a stepwise increase in the proportion of male patients (40.0%, 44.8%, 53.4%, *p* < 0.001), as well as age, level of fasting blood glucose, and Cr (all *p* < 0.001), and a stepwise decrease in eGFR (*p* = 0.046) and TC, LDL, and HDL level (*p* < 0.05) (Table 4).

**TABLE 4:** Baseline characteristics among three groups categorized by tertile of neutrophil count in the cohort study

	Overall	Lowest group	Middle group	Highest group	*p*
*Neutrophils*					
*n*	2060	687	733	640	
Age (years)	68.93 ± 0.13	68.87 ± 0.24	68.74 ± 0.21	69.22 ± 0.23	0.327
Gender (male %)	949 (46.1%)	292 (42.5%)	337 (46.0%)	320 (50.0%)^b^	0.024
BMI (kg/m^2^)	25.39 ± 0.07	25.00 ± 0.13	25.60 ± 0.13^a^	25.57 ± 0.13^b^	0.002
ALT (U/L)	19.0 (13.6–27.8)	18.8 (13.5–26.9)	19.6 (13.8–29.9)	18.9 (13.4–26.5)	0.403
TC (mmol/L)	5.11 ± 0.02	5.12 ± 0.04	5.17 ± 0.04	5.02 ± 0.04^c^	0.025
TG (mmol/L)	1.40 (1.00–2.03)	1.27 (0.94–1.86)	1.43 (1–1.97)^a^	1.54 (1.07–2.19)^b^	<0.001
HDL (mmol/L)	1.47 ± 0.01	1.53 ± 0.01	1.49 ± 0.01^a^	1.38 ± 0.01^b^ ^,^ ^c^	<0.001
LDL (mmol/L)	2.96 ± 0.01	2.93 ± 0.03	2.99 ± 0.03	2.95 ± 0.03	0.399
Cr (mol/L)	65.74 ± 0.41	64.06 ± 0.76	65.73 ± 0.63	67.54 ± 0.76^b^	0.004
eGFR (%)	101.41 ± 0.56	103.67 ± 0.99	100.81 ± 0.89^a^	99.66 ± 1.03^b^	0.012
FBG (mmol/L)	8.3 (7.4–10.1)	7.7 (7.1–9.5)	8.0 (7.3–9.3)	8.4 (7.5–10.9)^b^ ^,^ ^c^	<0.001
HbA1C (%)	7.6 (6.9–8.9)	7.1 (6.4–8)	7.2 (6.6–8.2)^a^	7.5 (6.7–8.6)^b^ ^,^ ^**c**^	<0.001
HBP (*n* %)	1238 (80.1%)	427 (76.7%)	470 (82.2%)^a^	341 (82.0%)^b^	0.037
*NLR*					
*n*	2060	682	692	686	
Age (years)	68.93 ± 0.13	68.33 ± 0.23	68.86 ± 0.22	69.59 ± 0.23^b^ ^,^ ^c^	0.001
Gender (male %)	949 (46.1%)	273 (40.0%)	310 (44.8%)	366 (53.4%)^b^ ^,^ ^c^	<0.001
BMI (kg/m^2^)	25.39 ± 0.07	25.53 ± 0.13	25.51 ± 0.13	25.13 ± 0.14	0.060
ALT (U/L)	19.0 (13.6–27.8)	19.1 (14.0–29.0)	19.7 (13.8–27.9)	18.1 (13.0–26.0)^b^	0.037
TC (mmol/L)	5.11 ± 0.02	5.28 ± 0.04	5.12 ± 0.03^a^	4.92 ± 0.03^b^ ^,^ ^c^	<0.001
TG (mmol/L)	1.40 (1.00–2.03)	1.43 (1.01–2.15)	1.49 (1.05–2.04)	1.31 (0.89–1.89)^b^ ^,^ ^c^	<0.001
HDL (mmol/L)	1.47 ± 0.01	1.51 ± 0.01	1.47 ± 0.01^a^	1.43 ± 0.01^b^ ^,^ ^c^	<0.001
LDL (mmol/L)	2.96 ± 0.01	3.05 ± 0.03	2.96 ± 0.03^a^	2.87 ± 0.03^b^ ^,^ ^c^	0.001
Cr (mol/L)	65.74 ± 0.41	63.52 ± 0.64	65.29 ± 0.67	68.38 ± 0.82^b^ ^,^ ^c^	<0.001
eGFR (%)	101.41 ± 0.56	103.21 ± 0.94	101.44 ± 0.92	99.58 ± 1.03^b^	0.031
FBG (mmol/L)	8.3 (7.4–10.1)	7.9 (7.1–9.3)	7.9 (7.2–9.5)	8.3 (7.4–10.4)^b^ ^,^ ^c^	<0.001
HbA1C (%)	7.6 (6.9–8.9)	7.2 (6.5–8.1)	7.3 (6.6–8.3)	7.2 (6.6–8.5)	0.435
HBP (*n* %)	1238 (80.1%)	455 (80.7%)	424 (80.2%)	359 (79.4%)	0.884

*Note*: Data are mean ± SE, median (interquartile range) or *n* (%).

Abbreviations: ALT, alanine transaminase; BMI, body mass index; Cr, creatinine; eGFR, estimated glomerular filtration rate; FBG, fasting blood glucose; HbA1C, glycosylated hemoglobin; HBP, high blood pressure; HDL, high‐density lipoprotein; LDL, low‐density lipoprotein; NLR, neutrophil‐to‐lymphocyte ratio; TC, total cholesterol; TG, triglycerides.

^a^ Middle group versus lowest group, *p* < 0.05.

^b^ Highest group versus lowest group, *p* < 0.05.

^c^ Highest group versus middle group, *p* < 0.05.

**FIGURE 4:**
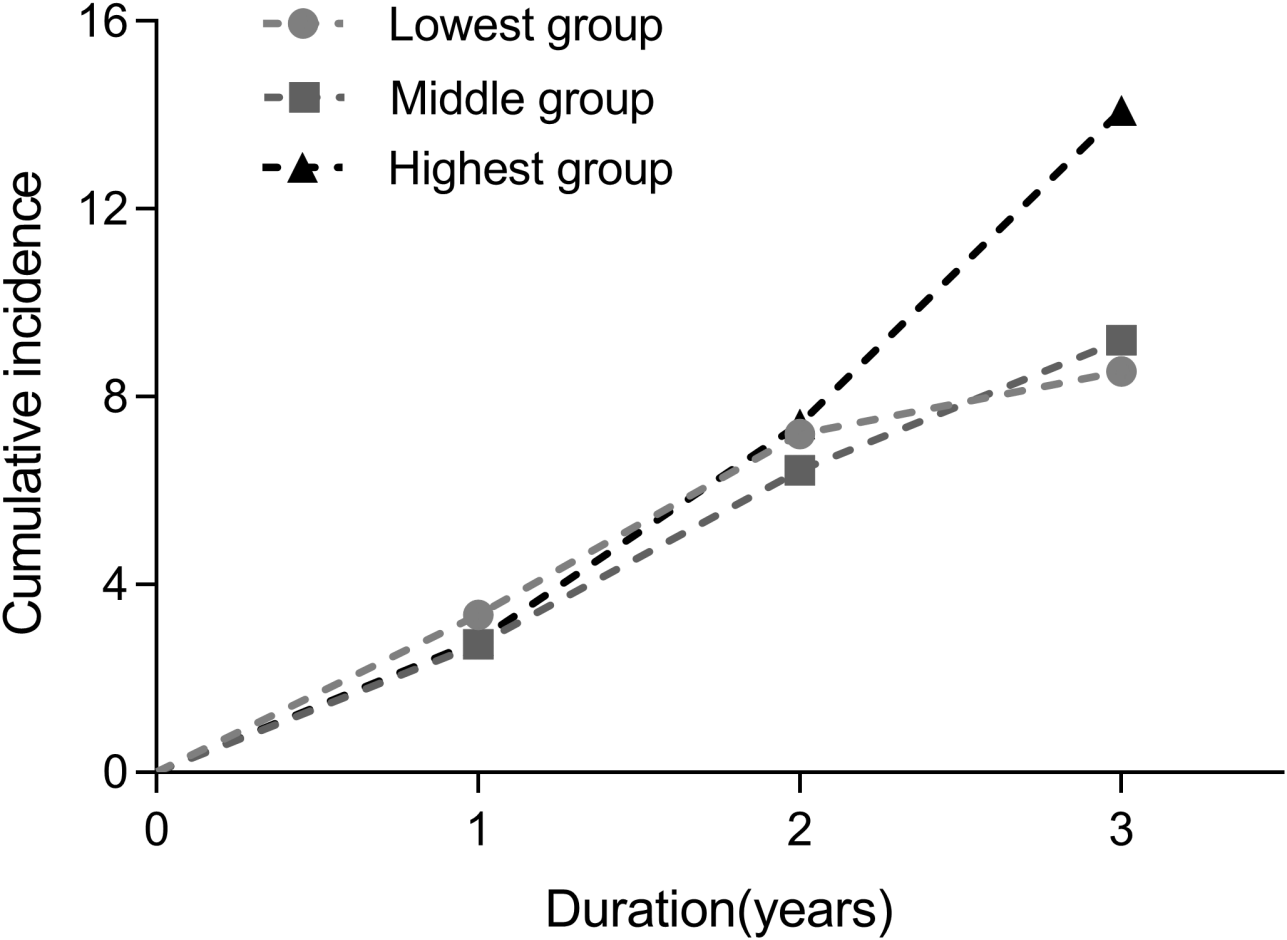
Cumulative incidence of nephropathy by tertile of neutrophil count in the cohort study. Lowest group (<3.19 × 10^9^/L), middle group (3.19–4.10 × 10^9^/L), and highest group (>4.10 × 10^9^/L). Emerged estimated glomerular filtration rate decreased by >30% in 53 (7.7%) subjects the lowest group, 60 (8.2%) subjects in the middle group, and 78 (12.2%) subjects in the highest group. *Log‐rank (Mantel‐Cox) test*: *p =* 0.031

Among the 2060 subjects with diabetes, there was an increasing incidence of eGFR decrease >30% in a graded manner with the increase of neutrophil tertile at baseline: eGFR decreased by more than 30% in 53 (7.7%) subjects in the lowest group, 60 (8.2%) subjects in the middle group, and 78 (12.2%) subjects in the highest group over the 4‐year follow‐up period. The same applied to NLR: eGFR decreased by more than 30% in 55 (7.8%) subjects in the lowest group, 65 (9.4%) in the middle group, and 73 (10.6%) in the highest group. In the crude model of neutrophils, hazard ratio (HR; 95% CI) for eGFR decrease >30% when comparing the highest group vs. the lowest group was 1.547 (1.091–2.193, *p* = 0.014). After adjusting for age, gender, and BMI, HR of neutrophil count for eGFR decrease >30% in the highest group remained significantly different compared with the lowest group 1.651 (1.146–2.378, *p* = 0.007). Finally, after fully adjusting for potential confounders, HR of neutrophil count for eGFR decrease >30% still showed a significant difference (the highest group vs. the lowest group, 2.293 [1.260–4.171], *p* = 0.007) (Table 5). The cumulative incidence of CKD gradually increased with increasing neutrophil count (Figure 4). Although Cox regression model of NLR was also implemented, the HR for eGFR decrease >30% showed no difference across the NLR tertiles (Table 5).

**TABLE 5:** Adjusted hazard ratios of incidence of estimated glomerular filtration rate decrease >30% in relation to tertile of neutrophil count among subjects with diabetes

	Incident case	Crude HR (95% CI)	*p*	Model 1	*p*	Model 2	*p*
*Neutrophils*							
Lowest group	53 (7.7%)	1.00 (reference)		1.00 (reference)		1.00 (reference)	
Middle group	60 (8.2%)	1.038 (0.717–1.502)	0.844	1.122 (0.766–1.643)	0.554	1.000 (0.524–1.908)	0.999
Highest group	78 (12.2%)	1.547 (1.091–2.193)	0.014	1.651 (1.146–2.378)	0.007	2.293 (1.260–4.171)	0.007
*NLR*							
Lowest group	55 (7.8%)	1.00 (reference)		1.00 (reference)		1.00 (reference)	
Middle group	65 (9.4%)	1.185 (0825–1.704)	0.358	1.234 (0.850–1.793)	0.269	1.358 (0.770–2.397)	0.291
Highest group	73 (10.6%)	1.327 (0.932–1.890)	0.117	1.466 (1.013–2.122)	0.043	1.415 (0.781–2.565)	0.252

*Note*: Model 1: adjustment for gender, age, BMI; model 2: model 1 plus adjustment for HbA1C, ALT, history of HBP, total cholesterol, triglyceride, HDL‐cholesterol, LDL‐cholesterol. Abbreviations: ALT, alanine transaminase; BMI, body mass index; CI, confidence interval; HbA1C, glycosylated hemoglobin; HBP, high blood pressure; HDL, high‐density lipoprotein; HR, hazard ratio; LDL, low‐density lipoprotein; NLR, neutrophil‐to‐lymphocyte ratio.

## 4 DISCUSSION

This cross‐sectional study and retrospective cohort study is the first with a large sample size to confirm an independent association of neutrophil count, not WBC, lymphocyte count or NLR, with the progression of CKD in patients with diabetes. A growing number of studies have described the crucial role of chronic inflammation in the development of CKD in patients with diabetes.^4^, ^5^, ^6^, ^12^, ^13^, ^14^ As the most important target of microvascular damage in diabetes, CKD is associated with both systemic and local renal inflammation with the participation of crucial inflammatory cells and molecules. As a simple indicator of inflammation, peripheral blood leukocytes comprising neutrophils, monocytes, and lymphocytes have previously been found to be related to the development of CKD in patients with diabetes.^7^, ^8^, ^9^, ^10^, ^15^ Our study suggested that of the peripheral blood leucocytes, neutrophil count was the most reliable independent risk factor for CKD, both in the cross‐sectional and cohort studies.

Neutrophil count and lymphocyte count are the principal components of the WBC count, and increasing WBC in CKD would have been largely due to the increased number of neutrophils. Because NLR is the ratio of neutrophil count to lymphocyte count, the increased NLR would likely have been due to increased neutrophils and decreased lymphocytes. As the largest fraction of white blood cells, neutrophils are involved in chronic meta‐inflammatory states such as obesity, insulin resistance, type 2 diabetes, gestational diabetes mellitus, and coronary artery disease.^10^, ^16^, ^17^, ^18^ Previous studies have revealed lymphocytes to be protective in coronary heart disease and heart failure.^19^, ^20^ Although our study and results from Chung et al^7^ both showed a decreased lymphocyte count, neither lymphocyte nor NLR were independent risk factors for CDK in patients with diabetes. It is proposed that the decreased lymphocyte count compensates for the increased neutrophils to maintain leukocyte homeostasis. Consequently, the study proved neutrophil count is most closely associated with CKD in patients with diabetes, not WBC, NLR, or lymphocyte count. This provides evidence of the important pathological role of innate immune cells in the development of CKD in patients with diabetes.

Increased neutrophils may participate in the occurrence and development of CKD in many ways. Studies from Mahfouz et al and Bolignano et al showed that neutrophil gelatinase‐associated lipocalin (NGAL) produced by neutrophils is a potential predictor of CKD and its progression,^21^, ^22^ although NGAL is not only synthesized by neutrophils; other cells are also involved in its synthesis such as epithelial cells.^23^ Other studies suggest that hyperglycemia promotes an increase in number of circulating neutrophils, and the migration of neutrophils through chemokines to the glomerular basement membrane injury site promotes the inflammatory cascade through further chemotaxis of mononuclear macrophages.^24^ Neutrophils may also contribute to CKD progression through neutrophil elastase (NE) that is secreted by activated neutrophils.^25^, ^26^ The proinflammatory effect of NE has been confirmed in a variety of disease models.^27^ Previous studies revealed that neutrophils contribute to the etiology of insulin resistance via secreted NE, which leads to the degradation of insulin receptor substrate 1.^27^, ^28^ NE also leads directly to renal cell damage, contributing to continuous progression of CKD in patients with type 2 diabetes.^29^ All these results demonstrate that neutrophils may contribute to CKD.

Our study also found that UA, advanced age, duration of diabetes, and history of hypertension were risk factors for CKD in patients with diabetes, consistent with previous studies.^1^, ^30^ It is clear that the progression of diabetic nephropathy is a complex process with many factors involved in the pathogenesis. Sembach et al reported no or only mild gender differences in the functional and structural changes during CKD progression in an animal study.^31^ On the contrary, in our study, female gender was an obvious risk factor for CKD in patients with diabetes. This may be due to hormonal changes in women after menopause and should be further verified in aged mice. We also found that advanced age was an independent risk factor for renal dysfunction, consistent with previous study demonstrating that eGFR declines in parallel with age.^32^, ^33^ Aging may therefore also be involved in the occurrence and development of CKD via its own mechanisms.

There were some limitations of this study. Although we have proved that among all white blood cells, neutrophils were the most predictive for CKD in patients with diabetes, the mechanism of the potentially pathophysiological role of neutrophils in the development of CKD has not been studied clinically. Further research in humans and in animal studies should be performed to clarify the function of neutrophil‐secreted proteins, especially NE and NGAL.

In conclusion, our study is the first to demonstrate the close association of neutrophil count with CKD in patients with diabetes. Neutrophil count was an independent risk factor for CKD especially when it exceeded 3.6 × 10^9^/L and increased neutrophil count could predict a faster decline of eGFR in patients with diabetes.

## FUNDING INFORMATION

Natural Science Research Funds of Minhang District, Shanghai (2019MHZ066). Health Profession Clinical Funds of Shanghai Municipal Health Commission (201940295). Scientific Research Project funded by Shanghai Fifth People's Hospital, Fudan University (2018WYZD04). Medical Key Faculty Foundation of Shanghai (ZK2019B15). Minhang District Health Committee Project (2020MW38).

## CONFLICT OF INTEREST

No potential conflicts of interest relevant to this article were reported.

## Supporting information


**FIGURE S1** Changes of neutrophils during 4 years among three groups categorized by tertile of neutrophil count in baseline. A for neutrophil count; B for neutrophil‐to‐lymphocyte ratio (NLR).

**TABLE S1** Changes of neutrophils and neutrophil‐to‐lymphocyte ratio (NLR) during 4 years among three groups categorized by tertile of neutrophil count in baseline
