# Supplementary figures and images for: Increased neutrophil count Is associated with the development of chronic kidney disease in patients with diabetes
Source: J Diabetes. 2022 Jul 4;14(7):442–54. doi: 10.1111/1753-0407.13292 (PMC9310049; doi:10.1111/1753-0407.13292)

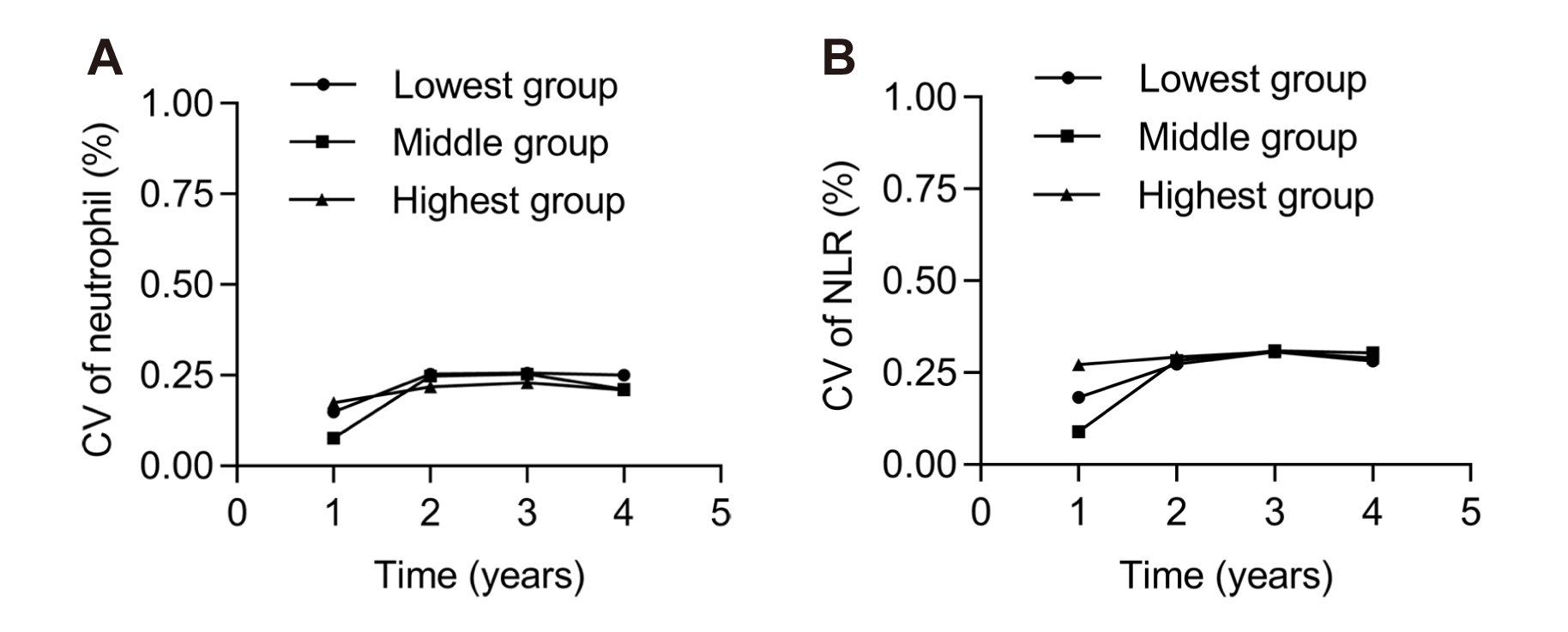

Supplement: Supplementary file 1 — FIGURE S1 Changes of neutrophils during 4 years among three groups categorized by tertile of neutrophil count in baseline. A for neutrophil count; B for neutrophil‐to‐lymphocyte ratio (NLR). [file JDB-14-442-s002.tif]
