# Supplementary material for: Increased neutrophil count Is associated with the development of chronic kidney disease in patients with diabetes
Source: J Diabetes. 2022 Jul 4;14(7):442–54. doi: 10.1111/1753-0407.13292 (PMC9310049; doi:10.1111/1753-0407.13292)
Supplement: Supplementary file 2 — TABLE S1 Changes of neutrophils and neutrophil‐to‐lymphocyte ratio (NLR) during 4 years among three groups categorized by tertile of neutrophil count in baseline [file JDB-14-442-s001.docx]

**Supplement Table 1. Changes of neutrophils and NLR during four years** **among three groups categorized by tertile of neutrophil count in baseline**

|  | Baseline | First year | Second year | Third year |
| --- | --- | --- | --- | --- |
| **Neutrophils** |  |  |  |  |
| **Mean ± SD** |  |  |  |  |
| Lowest group | 2.64±0.39 | 2.73±0.69 | 2.48±0.63 | 2.96±0.74 |
| Middle group | 3.62±0.27 | 3.22±0.80 | 3.33±0.84 | 3.50±0.74 |
| Highest group | 5.09±0.88 | 4.42±0.96 | 4.54±1.04 | 4.85±1.02 |
| ***CV* (%)** |  |  |  |  |
| Lowest group | 0.14 | 0.25 | 0.25 | 0.25 |
| Middle group | 0.07 | 0.24 | 0.25 | 0.21 |
| Highest group | 0.17 | 0.21 | 0.22 | 0.21 |
| **NLR** |  |  |  |  |
| **Mean ± SD** |  |  |  |  |
| Lowest group | 1.24±0.22 | 1.47±0.40 | 1.42±0.43 | 1.54±0.43 |
| Middle group | 1.84±0.16 | 1.74±0.49 | 1.85±0.57 | 1.91±0.58 |
| Highest group | 2.90±0.78 | 2.71±0.79 | 2.73±0.83 | 2.71±0.78 |
| ***CV* (%)** |  |  |  |  |
| Lowest group | 0.18 | 0.27 | 0.30 | 0.28 |
| Middle group | 0.09 | 0.28 | 0.30 | 0.30 |
| Highest group | 0.27 | 0.29 | 0.30 | 0.28 |

Abbreviations: CV, Coefficient of Variation; NLR, neutrophil-to-lymphocyte ratio.
